# Assessing the Relationship between Verbal and Nonverbal Cognitive Abilities Using Resting-State EEG Functional Connectivity

**DOI:** 10.3390/brainsci11010094

**Published:** 2021-01-13

**Authors:** Inna Feklicheva, Ilya Zakharov, Nadezda Chipeeva, Ekaterina Maslennikova, Svetlana Korobova, Timofey Adamovich, Victoria Ismatullina, Sergey Malykh

**Affiliations:** 1 Laboratory of Molecular Genetic Research of Human Health and Development, Scientific and Educational Center “Biomedical Technologies”, Higher Medical and Biological School, South Ural State University, 454080 Chelyabinsk, Russia; nadezda.chipeeva@ya.ru (N.C.); k.svetlana-1991@mail.ru (S.K.); 2 Developmental Behavioral Genetics Lab, Psychological Institute of Russian Academy of Education, 125009 Moscow, Russia; iliazaharov@gmail.com (I.Z.); tadamovich11@gmail.com (T.A.); victoria2686@gmail.com (V.I.); malykhsb@mail.ru (S.M.); 3 Center of Interdisciplinary Research in Education, Russian Academy of Education, 199121 Moscow, Russia; bayan-sulu@mail.ru

**Keywords:** functional connectivity of the brain, EEG, nonverbal and verbal cognitive abilities

## Abstract

The present study investigates the relationship between individual differences in verbal and non-verbal cognitive abilities and resting-state EEG network characteristics. We used a network neuroscience approach to analyze both large-scale topological characteristics of the whole brain as well as local brain network characteristics. The characteristic path length, modularity, and cluster coefficient for different EEG frequency bands (alpha, high and low; beta1 and beta2, and theta) were calculated to estimate large-scale topological integration and segregation properties of the brain networks. Betweenness centrality, nodal clustering coefficient, and local connectivity strength were calculated as local network characteristics. We showed that global network integration measures in the alpha band were positively correlated with non-verbal intelligence, especially with the more difficult part of the test (Raven’s total scores and E series), and the ability to operate with verbal information (the “Conclusions” verbal subtest). At the same time, individual differences in non-verbal intelligence (Raven’s total score and C series), and vocabulary subtest of the verbal intelligence tests, were negatively correlated with the network segregation measures. Our results show that resting-state EEG functional connectivity can reveal the functional architecture associated with an individual difference in cognitive performance.

## 1. Introduction

Individual differences in intelligence play a prominent role in human life. It largely determines success not only in learning but also in real-life outcomes, such as success in occupational or even marital status [1,2,3,4]. However, the neurophysiological mechanisms of intelligence are still understudied. Modern studies suggest that cognitive functions are the result of the concerted work of multiple brain structures [5,6,7]. The brain regions involved in cognition are connected to each other through anatomical and functional connections, forming networks [8]. The collaborative activity of different brain areas can be studied from the network neuroscience perspective. The use of a network neuroscience approach allows us to study the interaction of brain regions on the scale of the whole brain [9,10] and to identify global patterns of the brain networks activity underlying individual differences in cognitive abilities [11]. These global connectivity patterns can be revealed using mathematical graph theory. Herewith, if neurons or brain regions are represented as vertices of a network (graph), and synapses, neural connections, and/or temporal correlations of activity that may occur between pairs of brain regions—as the edges, the graph can be analyzed with specific metrics, characterizing its topological properties [12]. For example, these metrics can be used to describe fundamental processes within the brain: integration of brain networks (measured as the average or characteristic path length within the graphs, and segregation (measured as the coefficient of the clusterization and modularity of the graph [13,14].

According to the neural efficiency hypothesis of intelligence [15], a higher level of intelligence is characterized by efficient information flow in the brain (i.e., less energy spent). From the network neuroscience approach point of view, “efficiency” can be defined as cost minimization in the transfer of information within the network and is achieved through small-world network topology organization [16,17,18]. This form of the organization provides adequate information transmission with minimal power consumption and is characterized by a balance of integration and segregation processes in brain networks [19,20,21]. The neural efficiency hypothesis predicts that cognitive abilities will correlate with brain activity during cognitive load. However, whether the characteristics of brain efficiency, associated with individual differences in intelligence, can also be derived from EEG resting state is still unclear.

Previously, it has been shown that resting-state characteristics of brain activity can be a stable measure of the individual’s global functional connectivity [22,23]. The resting-state activity was associated with individual differences in several behavioral characteristics [24,25], including individual differences in the level of intelligence [26,27]. A study by Schultz and Cole [28] shows that more effective reconfiguration of functional connectivity at rest was associated with higher performance during cognitive tasks. According to Saxe and colleagues [29], there is a positive association between resting-state brain activity entropy and intelligence test performance, indicating that although the resting brain does not solve any specific tasks, resting-state activity creates prerequisites for more effective tasks solving in the future.

However, recent studies have failed to find an association between resting-state fMRI activity and various intelligence measures [30], while other studies showed that resting-state EEG network integration characteristics were positively correlated to individual differences in non-verbal intelligence [31,32]. It has been hypothesized that this lack of association between fMRI-resting states may be related to its poor temporal resolution (2–3 s [33]). In numerous EEG and MEG studies, it has been shown that high-frequency brain activity plays an important role in cognitive processes [34,35,36]. These results are also in line with a recent fast fMRI study [37] showing that different fMRI frequency bands (0.01–0.15 Hz, 0.15–0.37 Hz, 0.37–0.53 Hz, and 0.53–0.7 Hz) demonstrate band-specific shifts of the brain-wide neural coherence. The study by Nentwich et al. [38], however, showed that EEG functional connectivity patterns differ from fMRI connectivity patterns. These differences may be explained by the physiological origin of these two signals and the methods used to calculate functional connectivity.

The present study aimed to test whether EEG-resting state network characteristics are associated with verbal in addition to non-verbal cognitive abilities. Verbal intelligence refers to specific language skills and is evaluated by performing one or more special tests that include receptive and/or expressive speech, such as a vocabulary test, tasks to identify associations between words (verbal abstract reasoning), and providing factual answers to general knowledge questions [39]. Nonverbal abilities refer to “the ability to represent, transform, generate, and recall symbolic, non-linguistic information” [40]. According to the results of factor analysis, it is individual differences in verbal and nonverbal cognitive abilities that make the greatest contribution to individual differences in intelligence quotient [41], which is likely to make the neurobiological basis of verbal and non-verbal intelligence the most important of part of brain mechanism of general intelligence.

Previous fMRI-studies have shown the relationship between functional connectivity patterns with both nonverbal and verbal cognitive characteristics. For example, in the study by Pamplona and colleagues [42], the relationship between global characteristics of functional connectivity and verbal and non-verbal cognitive abilities were estimated. According to their study, the capacity of the “verbal comprehension” subscale (Wechsler Adult Intelligence Scale III) was positively correlated with global efficiency. Non-verbal cognitive abilities were significantly associated with local characteristics of functional connectivity, but not with global ones. EEG studies have shown that verbal abilities are associated with the EEG alpha power synchronization, especially in the anterior (from anteriofrontal to frontocentral) regions [43].

The current study aims to investigate whether both verbal and non-verbal individual differences characteristics are associated with large-scale topological properties of the brain networks and local functional connectivity characteristics according to the network neuroscience approach.

## 2. Materials and Methods

### 2.1. Participants

One hundred and forty-four students from universities and colleges in Moscow and Chelyabinsk aged 18 to 25 years (95 females) were recruited voluntarily to participate in the research. There were no monetary incentives for the participants.

All participants were assigned a unique ID and familiarized with the research procedure and gave their informed consent for inclusion before they participated in the study. The study was conducted in accordance with the Declaration of Helsinki, and the protocol was approved by the Ethics Committee for Interdisciplinary Research (protocol number 28122017-6 dated 23 December 2017). All participants had no history of neurological or mental disorders or head injuries. Twenty-nine participants were excluded from the analysis due to the lack of cognitive test data (16 people), and/or due to numerous excessive artifacts in EEG recording (more than 15% of the record, 13 people).

### 2.2. EEG Procedure

For EEG resting-state recording, we instructed all participants to sit calmly, not to think about anything specific, and not to fall asleep for 10 min. Every 2 min, participants were asked to open or close their eyes using the verbal instruction “Now open your eyes”, “Now close your eyes”, altogether with 6 min in eyes closed and 4 min in eyes open conditions. The EEG data with eyes closed only was used for analysis in this study. Verbal and nonverbal cognitive abilities were estimated online after recording EEG.

### 2.3. Intelligence Testing (Measures of Cognitive Abilities)

We applied the Raven’s Standard Progressive Matrices test [44] to estimate nonverbal cognitive abilities. The total amount of correct answers was used as a general indicator of nonverbal abilities, and the success of each of the five series (A, B, C, D, and E) was estimated separately. The series of the Raven’s “Standard Progressive Matrices” test were presented in the following order: A → B → C → D → E; each series was presented with increasing complexity. Participants were limited to 20 min to complete the test.

Verbal cognitive abilities were estimated using a short version of the vocabulary test “My Vocab” [45] and the verbal scales of the Universal Intellectual Test [46].

The short version of the vocabulary test consists of 95 words of different frequency of occurrence in the Russian language. The participants were asked to mark those words the meaning of which they knew. The volume of vocabulary was estimated by the number of words marked by the participant.

The Universal Intellectual Test (UIT) is a diagnostic complex for assessing the level and structure of intelligence. This test has high validity and reliability [47] and is widely used to evaluate intelligence in Russian samples. Universal Intellectual Test consists of 11 subtests, including nonverbal subtests (operating with figures and numbers) and verbal subtests (operating with words and verbal constructions). In our study, we used verbal subtests of the Universal Intellectual Test which measure the following parameters of intellectual functions: erudition (subtest “Awareness”) and deductive thinking, and the ability to operate with verbal information (subtest “Conclusions”). For each subtest, the number of correct answers was estimated. The subjects had a limited amount of time to complete the UIT subtests: the participants were given 6 min to complete the tests “Awareness” and 8 min for “Conclusions”.

### 2.4. EEG Data Acquisition and Pre-Processing

EEG data were recorded from 64 electrodes placed according to the international 10-10 system with a Brain Products ActiChamp amplifier (BrainProducts, Munich, Germany). All experiments were performed in a soundproofed and electrically shielded room with dim lighting. Impedance was maintained below 25 kOhm with a highly conductive chloride gel. The Brain Products PyCorder acquisition system was used for continuous recording without any filtering and continuous sampling at 500 Hz. The reference electrode was located at point Cz (re-referenced to average). The data were filtered from 0.1 to 30 Hz. Eyes closed/eyes open markers were added to separate continuous EEG into corresponding conditions. The “autoreject” algorithm for MNE was used for automatic artifact correction [48] was used. If the total length of the artifacts exceeded 15% of the overall recording, or if more than 2 electrode channels needed interpolation, the participant’s data was not included in the final analysis. After artifact correction, the data were divided into theta (4–8 Hz), alpha (full 8–13 Hz, low alpha—8–10 Hz, and high alpha 10–13 Hz), beta1 (13–20 Hz), and beta2 (20–30 Hz) frequency bands. To source reconstruction and functional connectivity analysis, the overall EEG recording was divided into 6-s epochs with a 1-s overlap to avoid the possible filter edge effects [49]. For every participant, around 60 epochs were used. 

### 2.5. Source Reconstruction

Functional connectivity was calculated for reconstructed sources of brain activity. Source reconstruction was performed for each frequency band using the standard source localization pipeline from MNE Python [50]. Firstly, a source space with 1026 sources per hemisphere was created. Secondly, the BEM (boundary-element model) method was used to create a three-layer model of the hemispheres: the inner skull, outer skull, and outer skin. The conductivity of layers was standard for the MNE package (0.3, 0.006, 0.3, respectively). The source was reconstructed using a standard model head (Colin27) without any individual anatomical images. Thirdly, the forward operator was constructed based on the source space and BEM model. And fourthly, the individual inverse operator was created for every participant with an individual noise covariance matrix. We used the standard BrainVision montage positions for the Electrical Lead Field estimation. The source reconstruction of each individual was performed with the appropriate inverse operator using a dSPM method. The Desikan–Killiany Atlas [51] was used for cortical parcellation with 34 ROI per hemisphere. Activation of the cortical ROI was extracted via the MNE function mne.extract_label_time_course with the “pca_flip” method. dSPM method of source reconstruction belongs to a minimum norm class of methods and is preferably used when distributed sources are expected. It is applied under the assumption that the currents constrained to the cortex and the amplitudes of the currents have a distribution with mean zero and a diagonal covariance matrix with equal variances for all sources. Though it is one of the most widely used source reconstruction methods, it can be substantially affected by the regularization procedure and is vulnerable to the creation of ghost sources.

### 2.6. Synchronization Measures

We used the built-in wPLI function from MNE Python [52,53] as a synchronization measure. The Weighted Phase Lag Index is a PLI extension that measures the asymmetry of the relative phase distribution. PLI ignores amplitude and is resistant to a false increase in coherence between signals due to common sources of brain activity. 

### 2.7. Graph Analysis

To calculate the graph, we constructed the adjacency matrices for every participant from synchronization estimates between electrical activities of pairs of anatomical structures resulting in a full weighted graph. The adjacency matrices were calculated separately for each epoch and then averaged before the network characteristics analysis. To avoid issues from the deliberate choice of threshold measure, we use the area under curve approach for each measure by the following procedure: each full graph was gradually thresholded starting from 10% of weakest connections with 10% step until top 10% remains. On each step, the graph measures were calculated. Then, for each measure, AUC was calculated on the values on each threshold. One of the problems of the connectivity analysis on the EEG data is the volume conduction effects. In the present study, we used the wPLI measure, designed to reduce the effect of common sources of brain activity on the connectivity measures. However, while wPLI is insensitive to zero-lag interactions, it is still prone to spurious interactions due to common input problems. To address this issue, we used a method developed by Shahbasi et al. [54]. The main idea of this method is to compare the connections on the real EEG data with surrogate data constructed as the superposition of independent EEG sources (the details of the method are described in [54]). The interactions found both in real and surrogate were not used in further connectivity analysis and graph metrics calculation. We calculated two types of graph measures in the present study. First, we calculated large-scale topological network metrics: the characteristic path length, modularity, and the averaged clustering coefficient. The characteristic path length is the shortest path length between all pairs of nodes; the clustering coefficient is the number of connections in the neighborhood of a certain node divided by the maximum number of possible connections between the neighbors of this node. Modularity is a measure of a division of a network into subgraphs (modules), which are characterized by more dense connections inside subgraphs than between them [19]. The characteristic path length is hypothesized to reflect the integration of the network. The clustering coefficient and modularity are hypothesized to be measures of network segregation [12].

We also calculated nodal (local) network characteristics. Nodal metrics included local connectivity strength, betweenness centrality, and nodal clustering coefficient. Betweenness centrality is a measure of all the shortest paths passed through a node; local connectivity strength is a sum of weights at all edges connected to a node. Nodal clustering coefficient is the proportion of existing nodal edges to possible edges. Both large-scale topological and local metrics were calculated using “network” package for Python [55]. Louvain method was used as the cluster detection algorithm.

### 2.8. Statistical Analysis

The descriptive analysis of all the variables (demographic data, connectivity metrics, cognitive abilities) collected was performed for each group (men and women). To study the relationship between large-scale topological functional connectivity metrics and characteristics of verbal and nonverbal cognitive abilities, we used Spearman’s correlation analysis. Correction for multiple comparisons was made using the FDR method [56]. To identify relationships between local functional connectivity characteristics and cognitive indicators, we used the Bayesian correlation approach [57] to reduce the probability of false-positive correlations due to multiple comparisons. The statistical processing was completed in the Statistical Programming Environment R [58] with packages “igraph”, “psych”, and “corrplot”.

## 3. Results

To test the comparability of the results between the two data collection labs, we applied Levene’s test for homogeneity of results. For all variables, the *p*-values were higher than 0.05 with no evidence to reject the null hypothesis about the equal variance between the groups. We calculated descriptive statistics for separately for cognitive variables and three types of connectivity metrics within narrow EEG frequency bands. The results are presented in Table 1, Table 2, Table 3 and Table 4.

**Figure 1:**
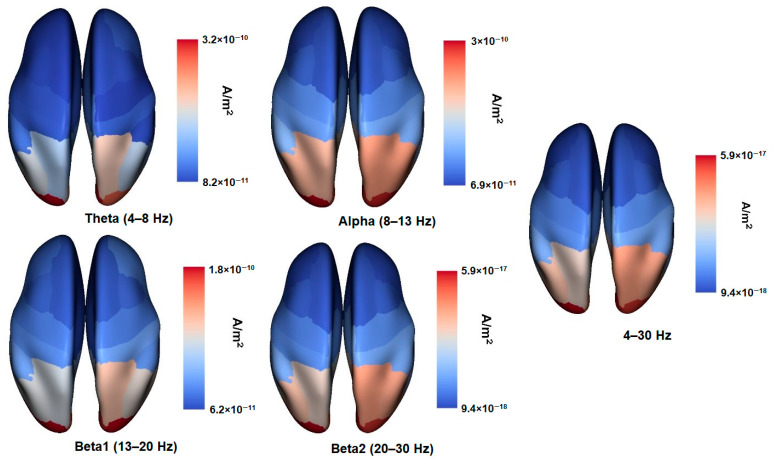
EEG sources density maps for theta (4–8 Hz), alpha (full 8–13 Hz), beta1 (13–20 Hz), beta2 (20–30 Hz), and 4–30 Hz frequency bands. 68 ROIs are reconstructed according to Desikan–Killiany Atlas [50]. Note that the density values are standardized for each frequency band individually.

The EEG source distribution maps for the Desikan Atlas ROIs are presented in Figure 1.

### 3.1. The Relationship between Verbal and Non-Verbal Abilities and Large-Scale Topological Network Characteristics

**Figure 2:**
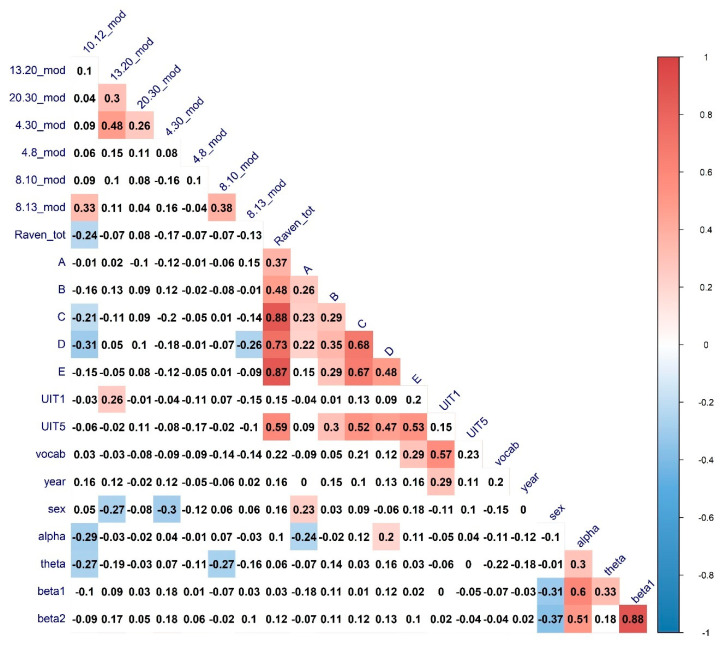
The heatmaps of the correlations between cognitive abilities measures, modularity, sex, age, and EEG Frequency bands power. Correlations of significance level *p* < 0.05 are marked in color. Mod—Modularity; UIT1—”Awareness” verbal subtest, UIT5—”Conclusion” subtest of the Universal Intellectual Test, vocab—“MyVocab” test. The results are adjusted for multiple comparisons with the FDR method.

**Figure 3:**
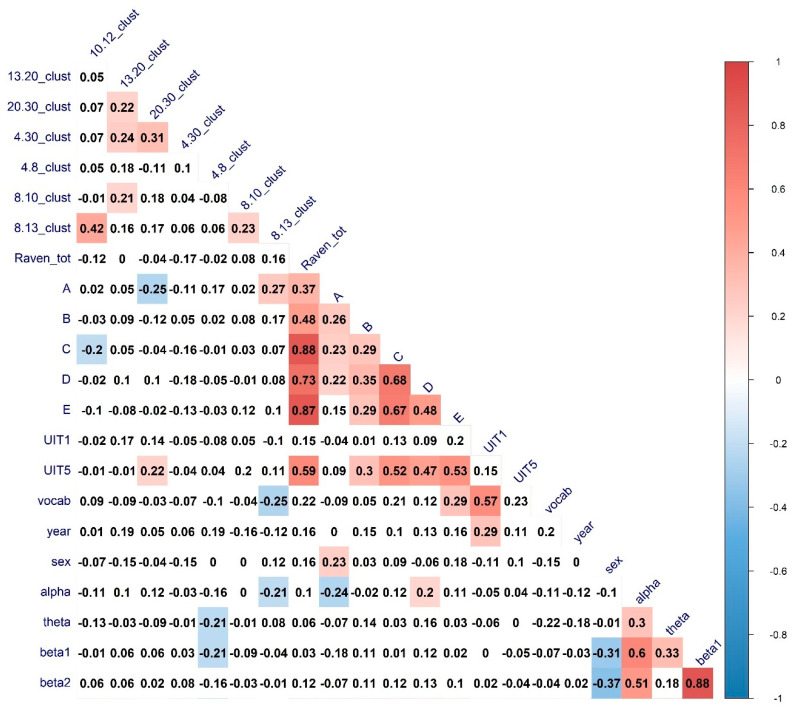
The heatmaps of the correlations between cognitive abilities measures, cluster coefficient, sex, age, and EEG Frequency bands power. Correlations of significance level *p* < 0.05 are marked in color. Clust—Coefficient of the clusterization; UIT1—“Awareness” verbal subtest, UIT5—“Conclusion” subtest of the Universal Intellectual Test, vocab—“MyVocab” test. The results are adjusted for multiple comparisons with the FDR method.

**Figure 4:**
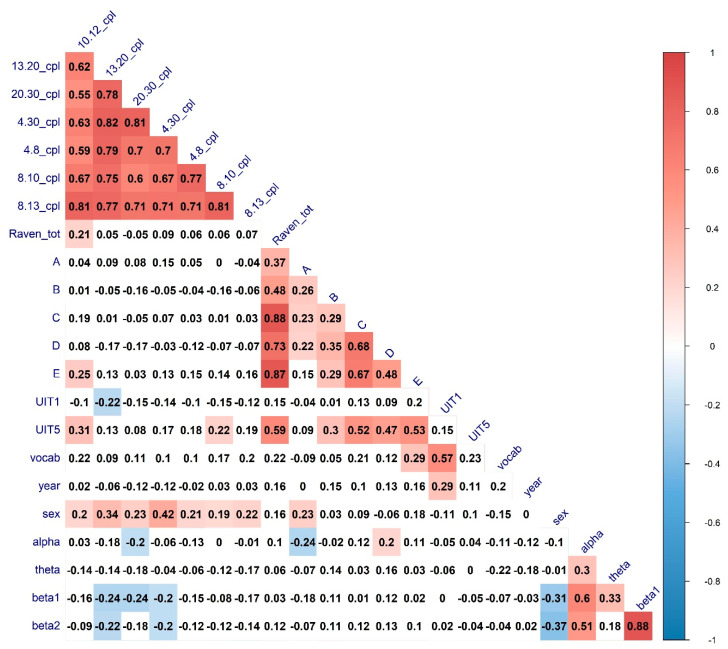
The heatmaps of the correlations between cognitive abilities measures, characteristic path length, sex, age, and EEG Frequency bands power. Correlations of significance level *p* < 0.05 are marked in color. CPL—Characteristic path length —Modularity; UIT1—“Awareness” verbal subtest, UIT5—“Conclusion” subtest of the Universal Intellectual Test, vocab—“MyVocab” test. The results are adjusted for multiple comparisons with the FDR method.

To investigate the complex interplay between the various connectivity metrics and the characteristics of verbal and nonverbal cognitive abilities, we have calculated the full zero-order correlation matrix of the interconnections between the cognitive variables and the connectivity graph measures in different frequency bands. The results of the Spearman correlations (FDR-corrected for multiple comparisons) are presented in Figure 2, Figure 3 and Figure 4.

It can be seen that for a number of topological properties, the sex of the participants and EEG band’s power were significant predictors. We ran separate partial correlations to regress out the effect of these variables. The results are presented in Table 5.

**Figure 5:**
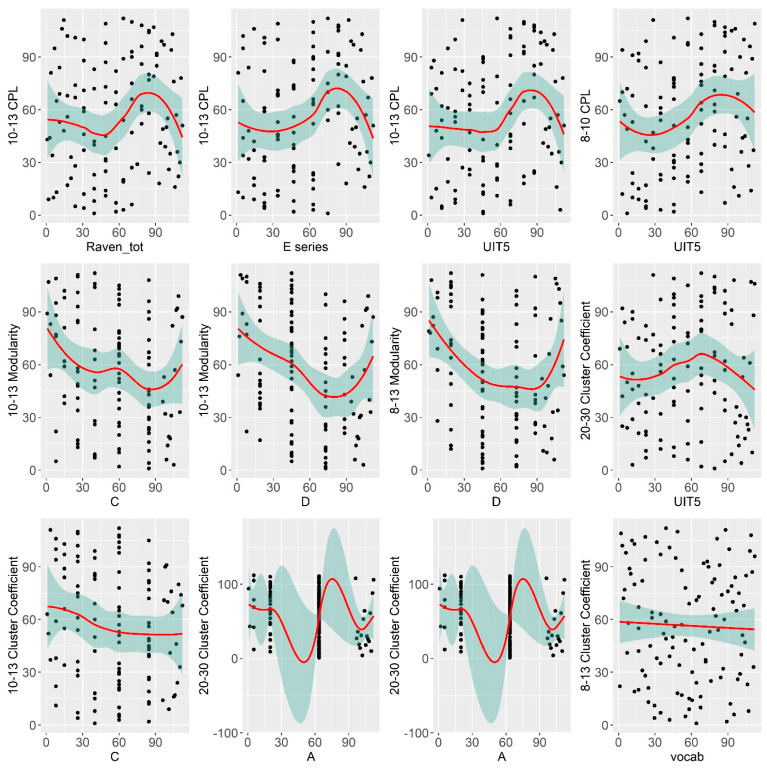
The scatterplots for the significant correlations. Correlations of significance level *p* < 0.05 are marked in color. CPL—Characteristic Path Length; UIT1—“Awareness” verbal subtest, UIT5—“Conclusion” subtest of the Universal Intellectual Test, vocab—“MyVocab” test. The ranks of the values were taken instead of the original value.

All the associations of interest except with the UIT1 (“Awareness”) test remained significant after partial correlation analysis. For the significant correlations, scatterplots with the linear trends have been calculated to visually inspect the relationship between the variables. The results are presented in Figure 5.

From the visual analysis of the scatterplots, it can be seen that correlations with the A and D Raven’s series are likely due to ceiling effects. Overall, it can be seen that both verbal and non-verbal cognitive abilities are correlated with integration and segregation network characteristics in the EEG alpha band (higher alpha, predominantly). To further investigate the relationship between alpha-band EEG network characteristics and cognitive abilities we calculated nodal integration and segregation connectivity metrics.

### 3.2. The Relationship between Verbal and Non-Verbal Abilities and Nodal Connectivity Measures

For each of the 68 reconstructed ROIs, we estimated the relationship between the given nodal connectivity characteristic and verbal and non-verbal cognitive measures. Numerous studies have shown that there are specific brain areas repetitively associated with intelligence. These brain areas have been combined into P-FIT (parieto-frontal integration) network [59]. We, thus, specifically tested whether nodal network characteristics of these areas were associated with verbal and non-verbal cognitive abilities in our study. The results are presented in Supplementary Materials Figures S1–S12 in the supplementary information.

Both positive and negative associations were found between betweenness centrality characteristics and cognitive measures. It can be seen that for the “MyVocab” test negative correlations are prevalent. For the “Awareness” subtest mostly positive correlations can be seen. There is no clear pattern for the involvement of the P-FIT areas. The highest correlations both for Raven’s total score and the Awareness subtest were found with the right pars triangularis ROI.

For the nodal cluster coefficient, it can be clearly seen that the association with local network segregation characteristics is opposite for verbal comparing to non-verbal cognitive abilities. For non-verbal intelligence, a lot of the highest correlation values are found for the P-FIT areas.

For the local connectivity strength characteristic, it can be seen that all the associations with both verbal and non-verbal characteristics are positive, however, very small and do not exceed *r* = 0.10.

## 4. Discussion

In our study, we were focused on the relationship between the large-scale topological brain network characteristics and local functional connectivity EEG measures during resting state and the level of verbal and nonverbal cognitive abilities. The local functional connectivity and the large-scale topological properties of the networks were estimated according to the network neuroscience approach [60]. We have found that network integration measures in the alpha band were positively correlated with non-verbal intelligence, especially with the more difficult part of the test (Raven’s total scores and E series). At the same time, individual differences in non-verbal intelligence (Raven’s total score and C series), and vocabulary subtest of the verbal intelligence tests, were negatively correlated with the whole-brain segregation measures. It should be noted that the association between network characteristics and individual differences in cognition found in the present study does not exceed the *r* = 0.3, which is quite a moderate correlation. However, according to the meta-analysis by Gignac [61], such a correlation value should be considered typical for the individual differences research.

The negative association between segregation and cognition is contrary to the previous results by Langer et al. [31] who showed positive relationships between the degree of segregation of the global brain network and nonverbal cognitive abilities. However, these results are in line with the previous results by Zakharov et al. [32], who showed that the alpha band characteristic path length of the brain graphs are robustly associated with non-verbal intelligence across different connectivity calculation routines. The present results are also consistent with structural brain connectivity data [62], which showed that high density and low modularity of white matter fibers are associated with higher fluid intelligence. Note, however, that anatomical and functional connectivity results should be compared with caution [38]. Previously, it has been shown that brain integration increases during cognitive load [14]. Our results may indicate that segregation decrease in a situation without any specific cognitive demand at rest with eyes closed [63], can provide the brain with the resources to higher performance and more efficient transmission of information during high-demand test administration.

Resting-state connectivity characteristics analyzed in our study can also capture the intrinsic frequency-specific brain functional architecture of the communication dynamics within the brain [34,60]. In the present study, we found the association between individual differences in cognitive abilities and the network characteristics in the EEG alpha range. EEG alpha power has been associated with numerous brain state characteristics that can play an important role in cognition, e.g., vigilance [64,65] or inhibition [66]. Individual differences in the alpha band were also shown to be related to giftedness and creativity [67] and language abilities [68]. It has also been directly associated with intelligence [43,69], though the results are mixed.

According to our results, the association between local network characteristics and verbal and non-verbal cognition are generally in line with the P-FIT theory of intelligence [59]. We have found that verbal and non-verbal intelligence performance are inversely correlated with the local brain segregation characteristics. These results are consistent with data from an fMRI study, which shows that at rest, the regional homogeneity of the regions included in P-FIT positively correlates with the level of cognitive abilities [70]. However, the results of the local brain source activation analysis derived from EEG should be interpreted with caution given the minimum norm methods constraints and the fact that it was based on the standard head template without individual MRI-data.

### Limitations

Firstly, in our study, we evaluated the relationship of only certain aspects of verbal and nonverbal cognitive abilities with the characteristics of functional connectivity of the brain at rest. In future studies, we are planning to expand the number of cognitive areas tested. Secondly, in the present study, the connectivity metrics were calculated for EEG sources reconstructed from the standard head model, which can bias the results. The individual EEG-MRI data are needed to confirm the results of the present study. Thirdly, our research sample consisted of young adults only. Further research is needed to understand whether the results we found can be generalized to other age ranges.

## 5. Conclusions

To sum up, we have found that both verbal and non-verbal cognitive abilities are associated with the local and the large-scale topological characteristics of the brain networks in the EEG alpha band. Our results show that resting-state brain can reveal the functional architecture that are associated with the individual difference in cognitive performance. The relationship between resting-state and on-task brain network characteristics and the reorganization of the brain networks are needed to better understand the neurophysiological basis of intelligence according to the network neuroscience approach.

## Figures and Tables

**Table 1:** Descriptive statistics of cognitive variables.

	Mean	Sd	Median	Min	Max	Skew	Kurtosis
Total	49.89	6.59	51.00	21.00	60.00	−1.47	3.27
A	11.47	1.24	12.00	2.00	12.00	−5.19	34.21
B	11.07	1.45	11.00	2.00	12.00	−3.32	15.86
C	9.99	1.87	11.00	3.00	12.00	−1.05	0.99
D	9.89	1.59	10.00	3.00	12.00	−1.52	3.82
E	7.59	2.53	8.00	2.00	12.00	−0.36	−0.53
Vocab	65.84	15.27	67.00	19.00	92.00	−0.76	0.55
Aw	8.92	3.37	9.00	0.00	15.00	−0.53	0.34
Conc	10.67	3.59	11.00	0.00	16.00	−0.71	−0.11

A, B, C, D, E—scores of the series of the Raven test, total—total score on the Raven test, Vocab—scores of the Vocabulary test, Aw—scores of the verbal subtest “Awareness”, Conc—scores of the verbal subtest “Conclusion”.

**Table 2:** Descriptive statistics of characteristic path length metric for narrow EEG bands.

EEG Band	Mean	Sd	Median	Min	Max	Skew	Kurtosis
10–12 Hz	0.27	0.06	0.25	0.18	0.42	0.83	−0.16
13–20 Hz	0.26	0.06	0.24	0.19	0.44	0.84	−0.09
20–30 Hz	0.27	0.06	0.25	0.19	0.46	0.86	0.05
4–30 Hz	0.27	0.06	0.25	0.19	0.44	0.79	−0.22
4–8 Hz	0.26	0.06	0.25	0.18	0.42	0.8	−0.25
8–10 Hz	0.26	0.06	0.25	0.18	0.42	0.74	−0.21
8–13 Hz	0.27	0.06	0.25	0.19	0.43	0.77	−0.27

**Table 3:** Descriptive statistics of cluster coefficient metric for narrow EEG bands.

EEG Band	Mean	Sd	Median	Min	Max	Skew	Kurtosis
10–12 Hz	0.19	0.03	0.19	0.13	0.40	2.46	15.20
13–20 Hz	0.26	0.03	0.27	0.17	0.37	−0.59	1.20
20–30 Hz	0.27	0.04	0.28	0.13	0.40	−0.90	2.31
4–30 Hz	0.32	0.05	0.33	0.18	0.41	−1.13	0.99
4–8 Hz	0.24	0.03	0.24	0.16	0.39	1.46	12.04
8–10 Hz	0.20	0.03	0.20	0.14	0.39	2.50	16.21
8–13 Hz	0.23	0.03	0.23	0.14	0.38	0.48	3.55

**Table 4:** Descriptive statistics of modularity metric for narrow EEG bands.

EEG Band	Mean	Sd	Median	Min	Max	Skew	Kurtosis
10–12 Hz	0.10	0.01	0.10	0.07	0.13	0.22	−0.05
13–20 Hz	0.09	0.01	0.09	0.07	0.12	0.72	0.36
20–30 Hz	0.09	0.01	0.09	0.06	0.13	0.60	0.93
4–30 Hz	0.09	0.01	0.09	0.07	0.12	0.61	0.65
4–8 Hz	0.09	0.01	0.09	0.06	0.12	0.43	1.22
8–10 Hz	0.10	0.01	0.09	0.07	0.13	0.53	−0.31
8–13 Hz	0.09	0.01	0.09	0.06	0.11	0.01	−0.44

**Table 5:** The results of the partial correlation analysis between the EEG measures, verbal and non-verbal cognitive variables, and potential regressors.

Variable	Correlate	Regressor	R Estimate	*p*-Value
Raven total	10–12 CPL	Sex	0.21 *	0.04
Raven total	10–12 Modularity	Alpha & Theta power	−0.24 *	0.02
A series	8–13 Clust. Coef.	Alpha power	0.16	0.11
C series	10–12 Modularity	Alpha & Theta power	−0.22 *	0.02
D series	10–12 Modularity	Alpha & Theta power	−0.26 *	0.01
E series	10–12 CPL	Sex	0.22 *	0.03
UIT1	13–20 CPL	Sex	−0.02	0.8
UIT1	13–20 Mod	Sex	0.13	0.2
UIT5	10–12 CPL	Sex	0.28 **	0.005
UIT5	8–10 CPL	Sex	0.28 **	0.006
MyVocab	8–13 Clust. Coef.	Alpha power	−0.29 **	0.001

CPL—Characteristic Path Length, Clust. Coef—Cluster Coefficient, Mod—Modularity; UIT1—”Awareness” verbal subtest, UIT5—”Conclusion” subtest of the Universal Intellectual Test, vocab—“MyVocab” test. * *p* < 0.05. ** *p* < 0.01.

## Data Availability

The data presented in this study are available on request from the corresponding author. The data are not publicly available due to data privacy regulations.

## Supplementary Materials

The following are available online at https://www.mdpi.com/2076-3425/11/1/94/s1, Figure S1: The relationship between non-verbal abilities (the Raven test (total score) and betweennes centrality of the nodes of the brain network, Figure S2: The relationship between verbal abilities (the test UIT1 (“Awareness”) and betweennes centrality of the nodes of the brain network, Figure S3: The relationship between verbal abilities (test UIT5 (“Conclusions”) and betweennes centrality of the nodes of the brain network, Figure S4: The relationship between verbal abilities (the test MyVocab) and betweennes centrality of the nodes of the brain network, Figure S5: The relationship between non-verbal abilities (Raven test (total score) and nodal clustering coefficient of the brain network, Figure S6: The relationship between verbal abilities (the test UIT1 (“Awareness”) and nodal clustering coefficient of the brain network, Figure S7: The relationship between verbal abilities (the test UIT5 (“Conclusions”) and nodal clustering coefficient of the brain network, Figure S8: The relationship between verbal abilities (the test MyVocab) and nodal clustering coefficient of the brain network, Figure S9: The relationship between non-verbal abilities (Raven test (total score) and local connectivity strengths of the nodes in the brain network, Figure S10: The relationship between verbal abilities (the test UIT1 (“Awareness”) and local connectivity strengths of the nodes in the brain network, Figure S11: The relationship between verbal abilities (the test UIT5 (“Conclusions”) and local connectivity strengths of the nodes in the brain network, Figure S12: The relationship between verbal abilities (the test MyVocab) and local connectivity strengths of the nodes in the brain network.
