# Supplementary material for: Assessing the Relationship between Verbal and Nonverbal Cognitive Abilities Using Resting-State EEG Functional Connectivity
Source: Brain Sci. 2021 Jan 13;11(1):94. doi: 10.3390/brainsci11010094 (PMC7828310; doi:10.3390/brainsci11010094)
Supplement: Supplementary file 1 [file brainsci-11-00094-s001.pdf]

Supplementary Materials

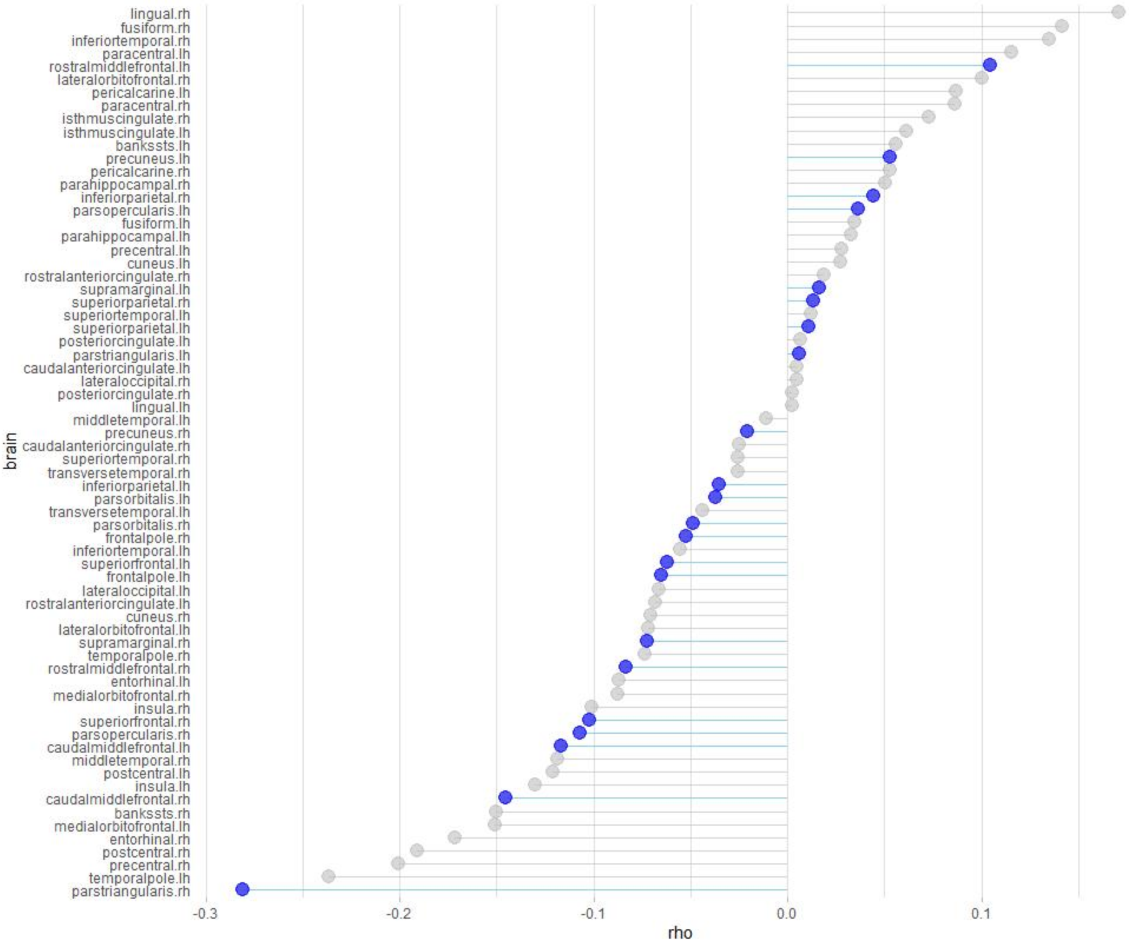

Figure S1. The relationship between non-verbal abilities (the Raven test (total score) and betweennes centrality of the nodes of the brain network. 68 ROIs are reconstructed according to Desikan-Killiany Atlas [50]. The correlations are sorted from the highest to lowest. The P-FIT areas are highlighted with blue.

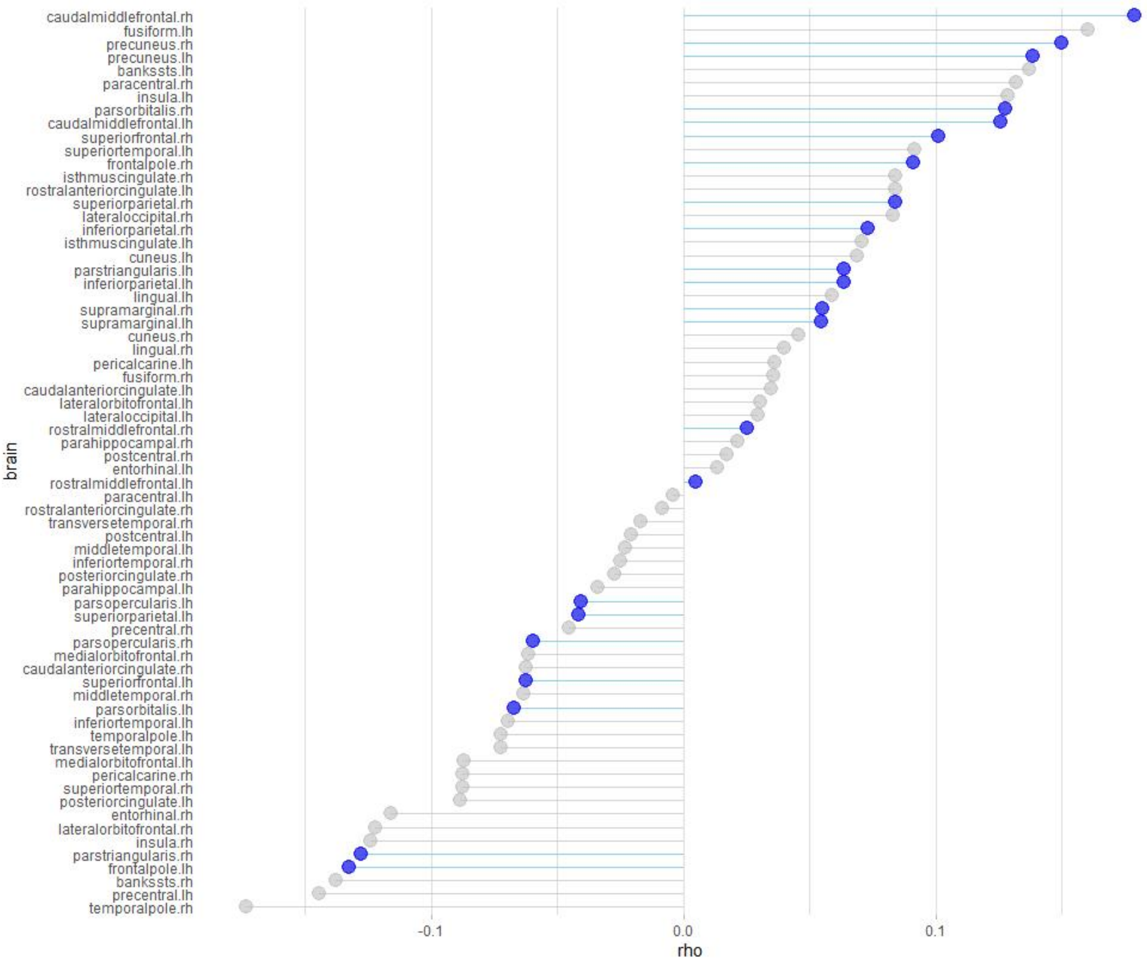

Figure S2. The relationship between verbal abilities (the test UIT1 ("Awareness") and betweennes centrality of the nodes of the brain network. 68 ROIs are reconstructed according to Desikan-Killiany Atlas [50]. The correlations are sorted from the highest to lowest. The P-FIT areas are highlighted with blue.

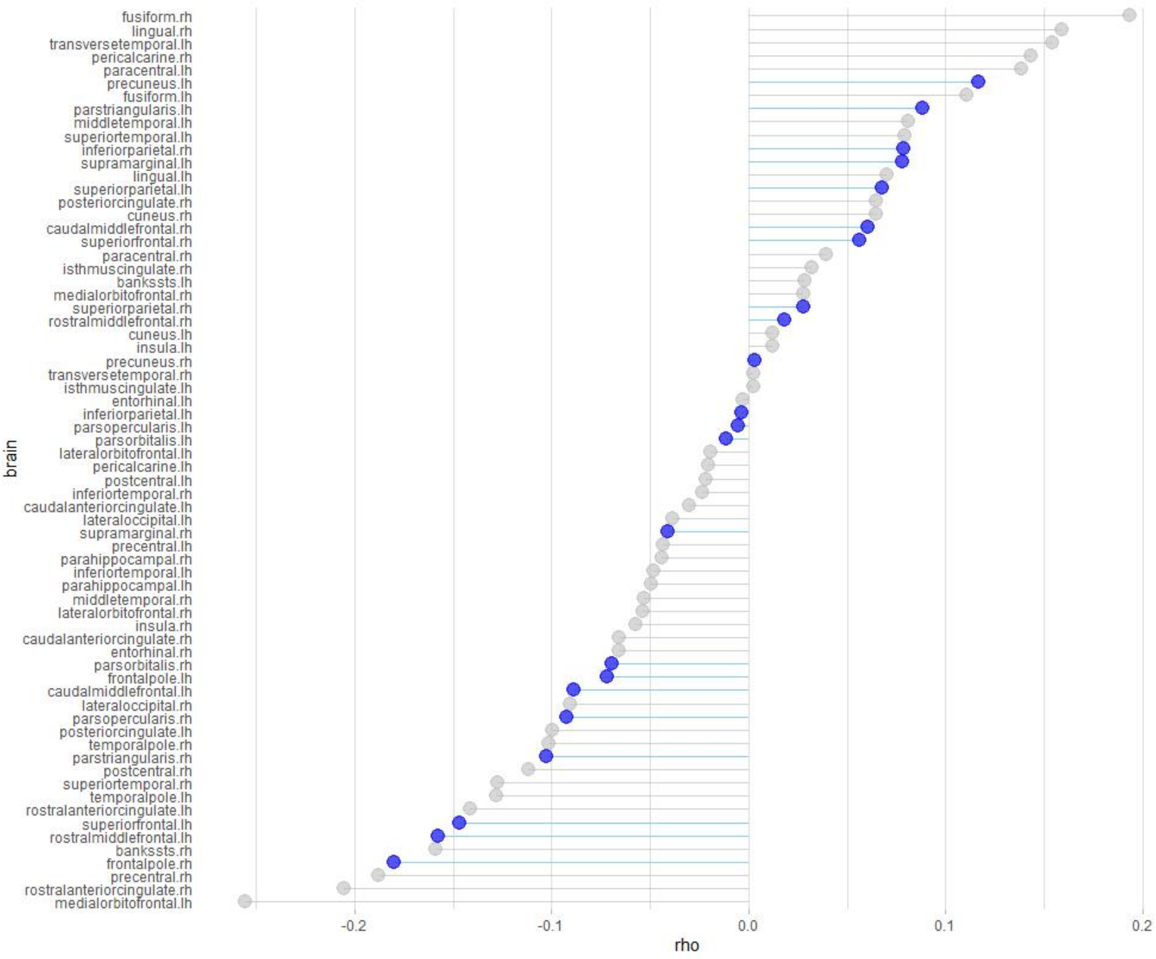

Figure S3. The relationship between verbal abilities (test UIT5 ("Conclusions") and betweennes centrality of the nodes of the brain network. 68 ROIs are reconstructed according to Desikan-Killiany Atlas [50]. The correlations are sorted from the highest to lowest. The P-FIT areas are highlighted with blue.

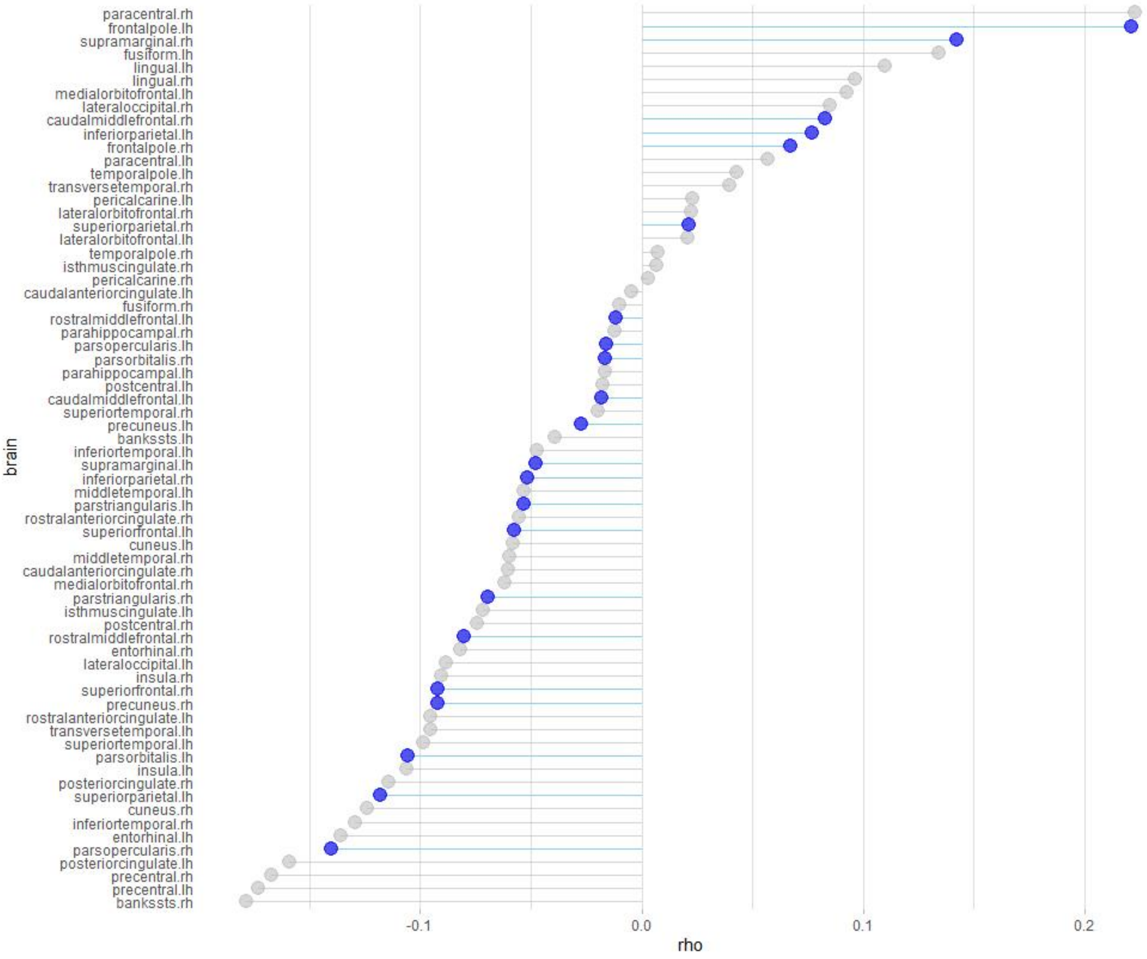

Figure S4. The relationship between verbal abilities (the test MyVocab) and betweennes centrality of the nodes of the brain network. 68 ROIs are reconstructed according to Desikan-Killiany Atlas [50]. The correlations are sorted from the highest to lowest. The P-FIT areas are highlighted with blue.

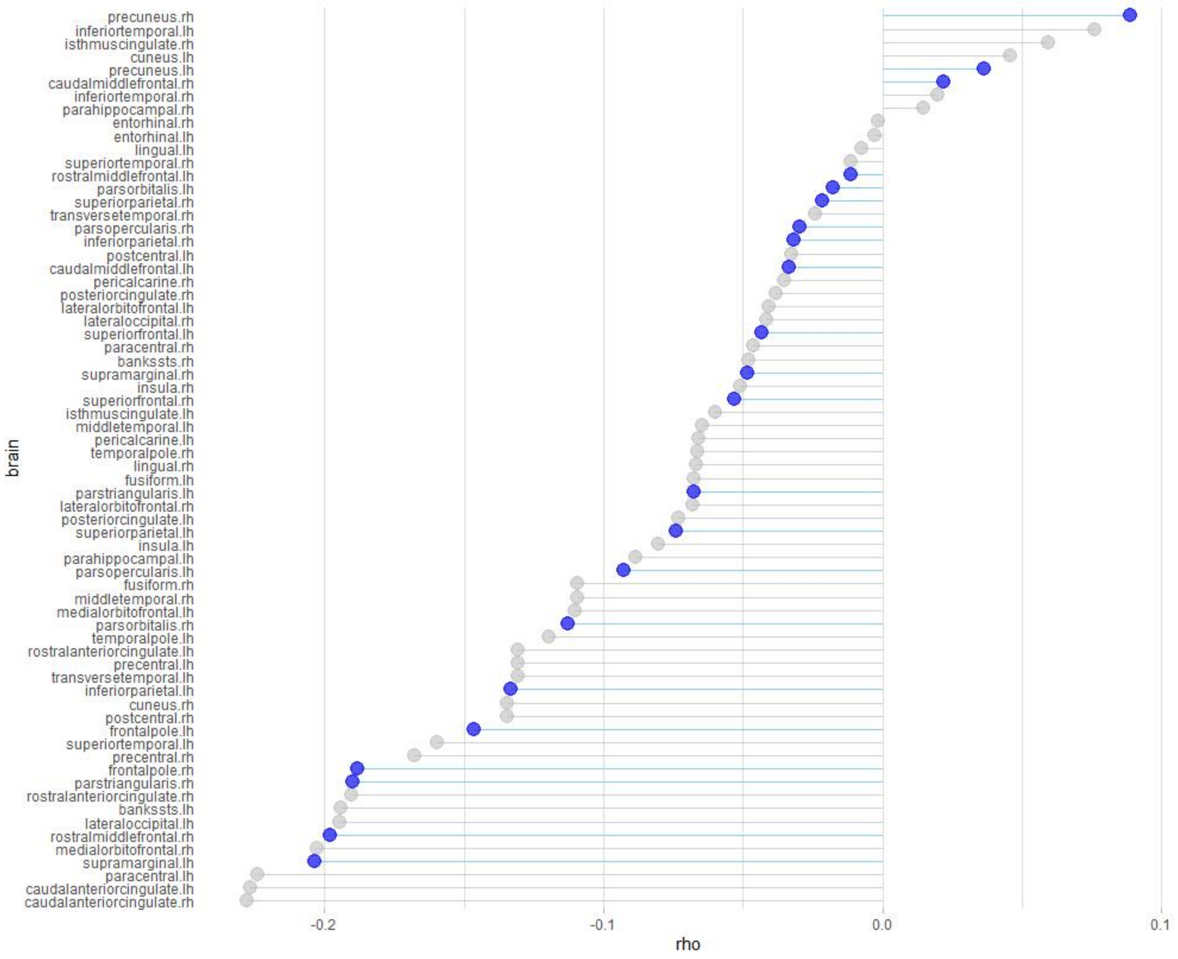

Figure S5. The relationship between non-verbal abilities (Raven test (total score) and nodal clustering coefficient of the brain network. 68 ROIs are reconstructed according to Desikan-Killiany Atlas [50]. The correlations are sorted from the highest to lowest. The P-FIT areas are highlighted with blue.

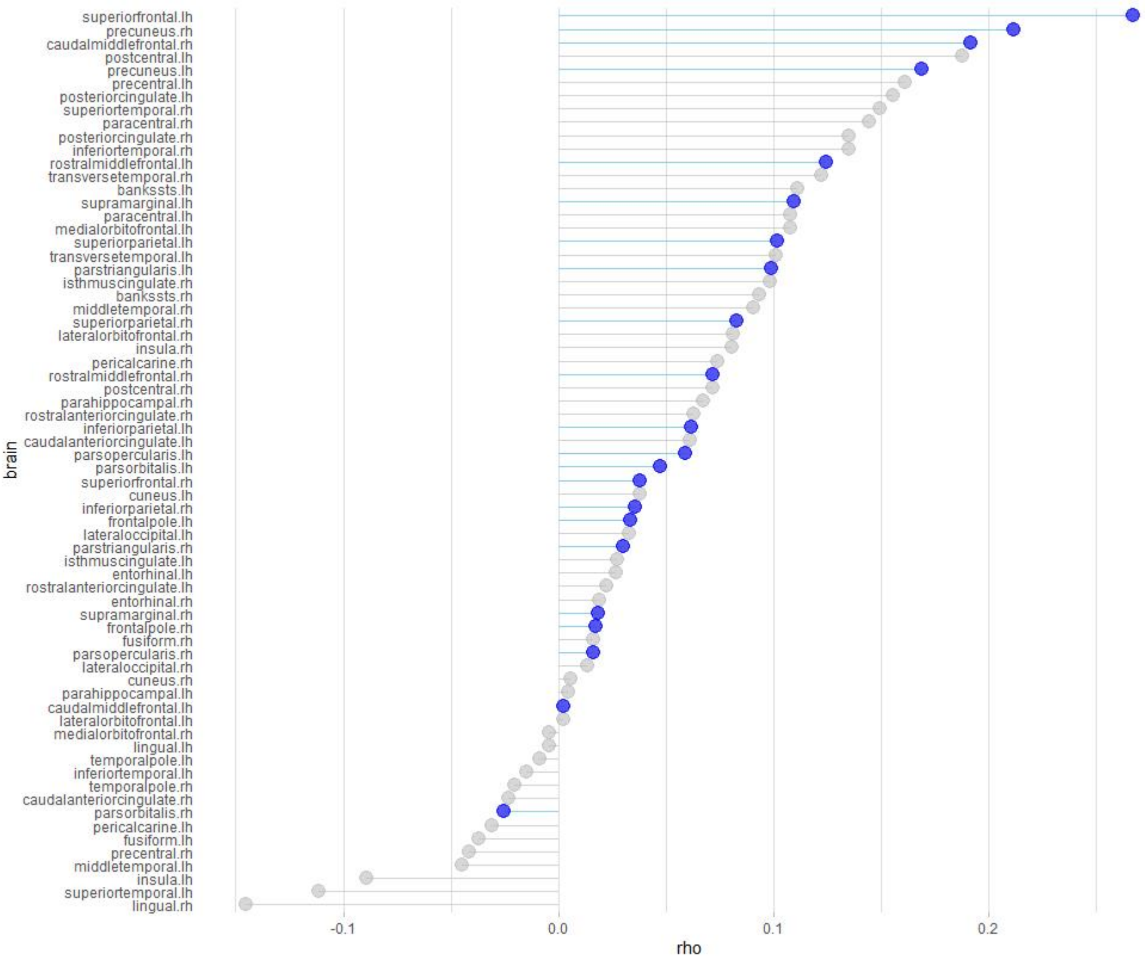

Figure S6. The relationship between verbal abilities (the test UIT1 ("Awareness") and nodal clustering coefficient of the brain network. 68 ROIs are reconstructed according to Desikan-Killiany Atlas [50]. The correlations are sorted from the highest to lowest. The P-FIT areas are highlighted with blue.

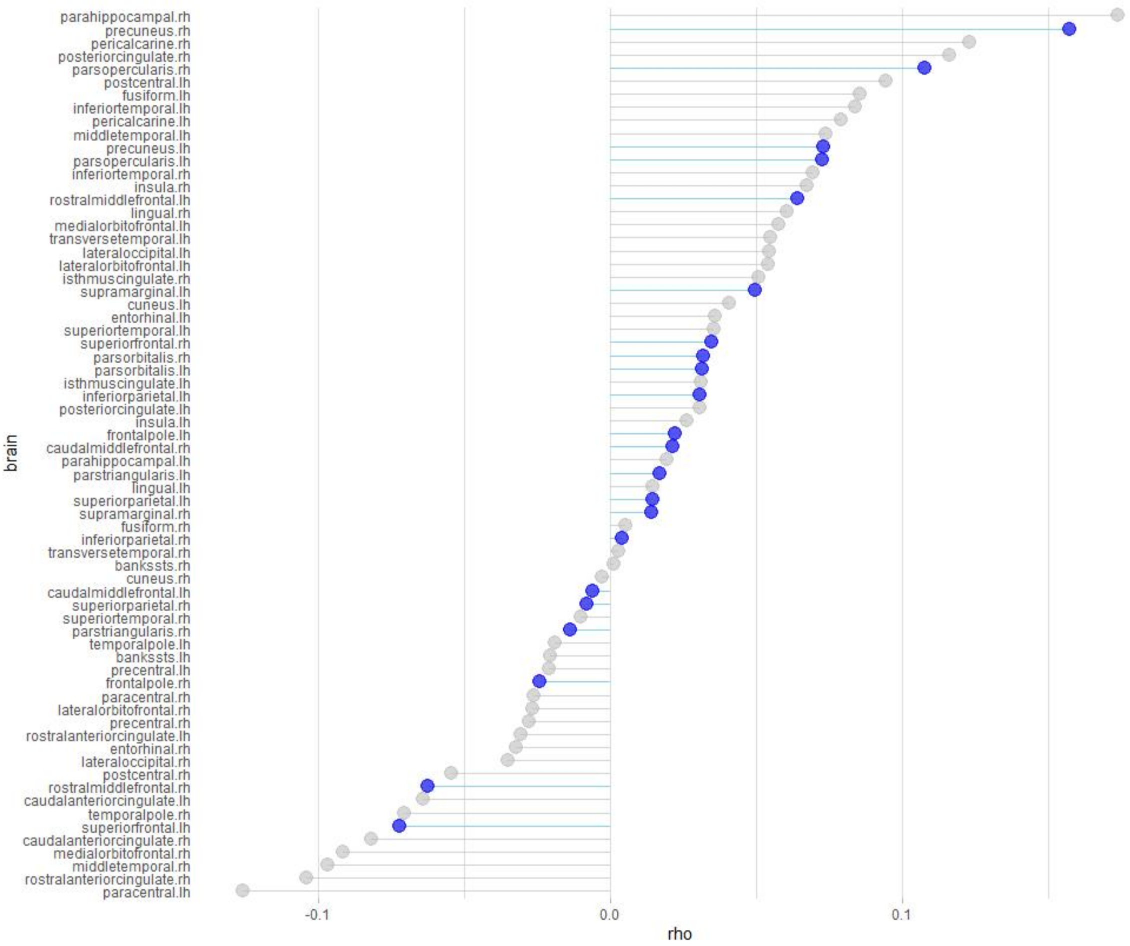

Figure S7. The relationship between verbal abilities (the test UIT5 ("Conclusions") and nodal clustering coefficient of the brain network. 68 ROIs are reconstructed according to Desikan-Killiany Atlas [50]. The correlations are sorted from the highest to lowest. The P-FIT areas are highlighted with blue.

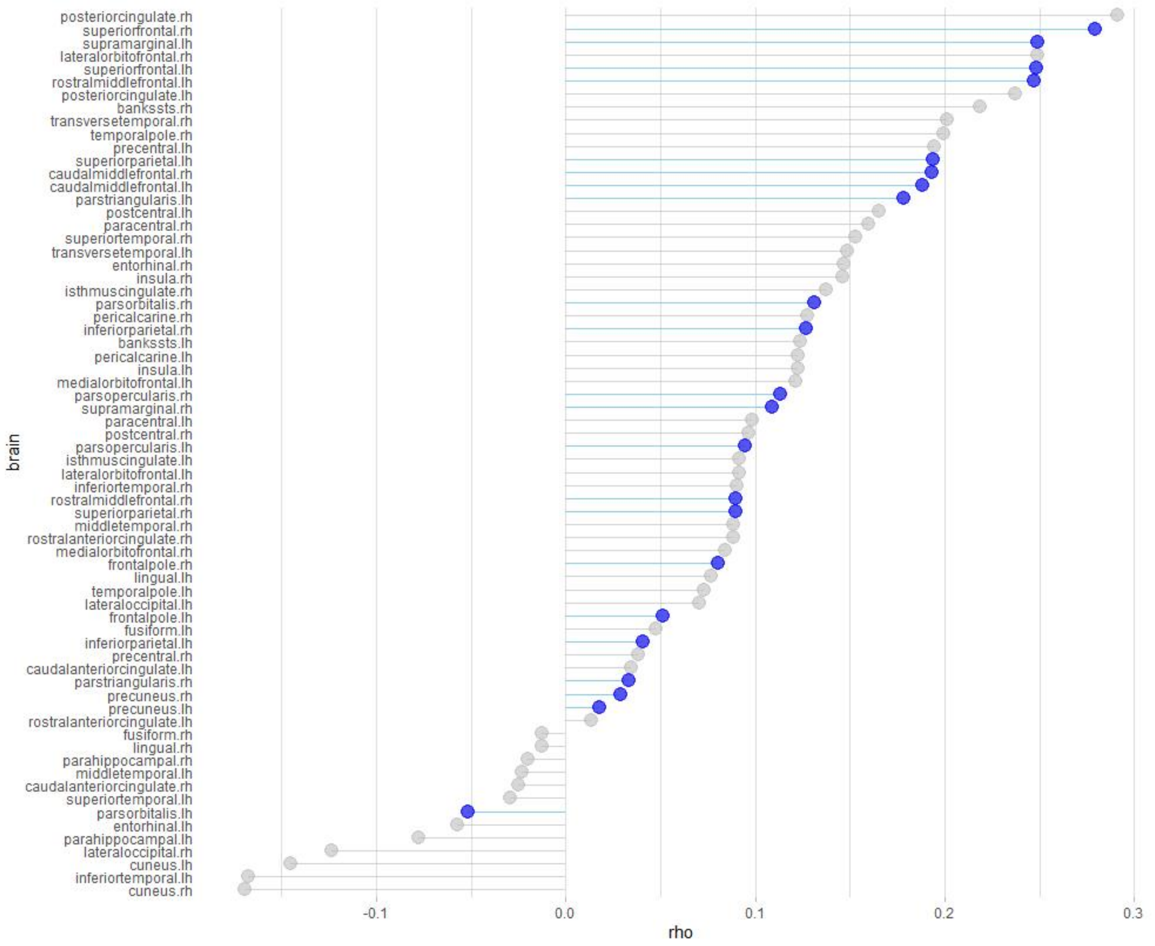

Figure S8. The relationship between verbal abilities (the test MyVocab) and nodal clustering coefficient of the brain network. 68 ROIs are reconstructed according to Desikan-Killiany Atlas [50]. The correlations are sorted from the highest to lowest. The P-FIT areas are highlighted with blue.

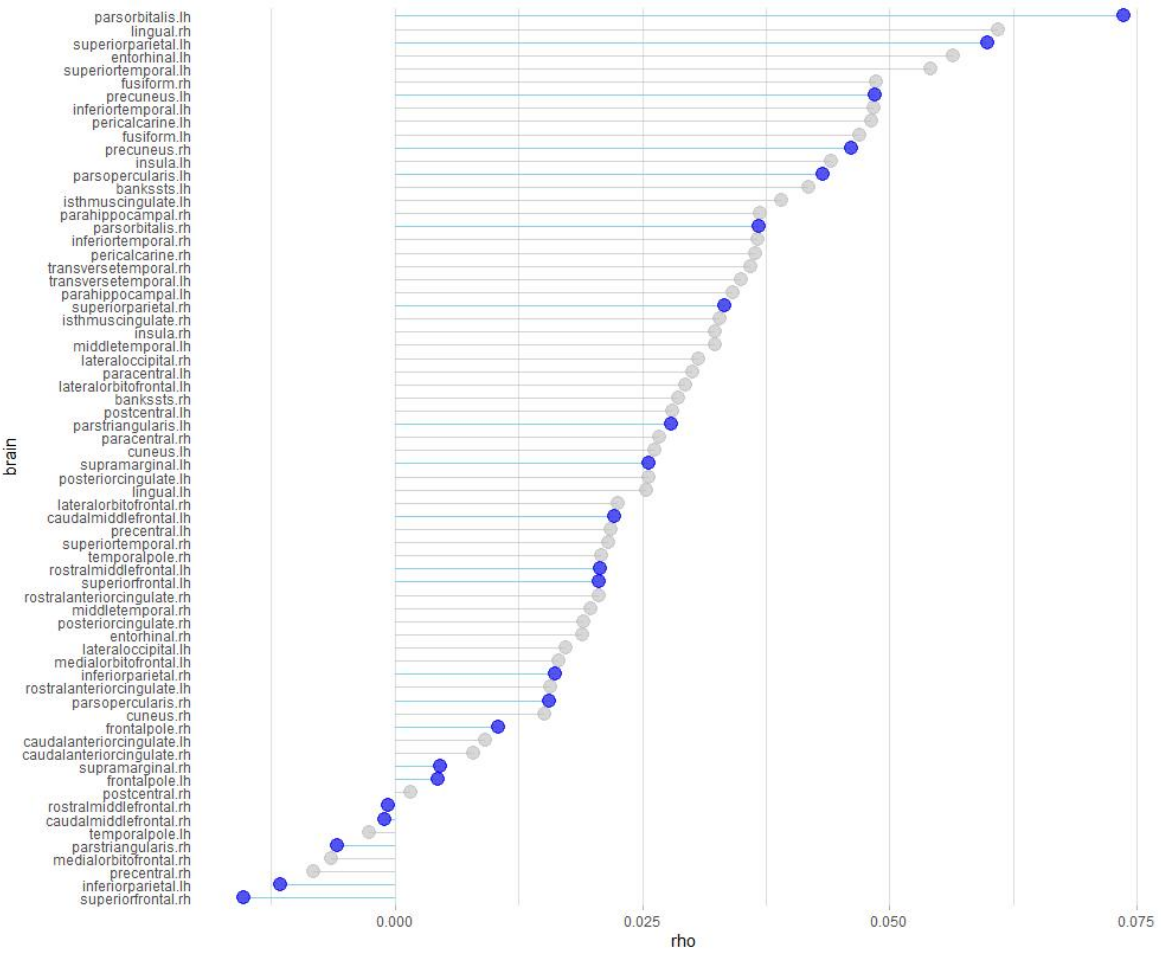

Figure S9. The relationship between non-verbal abilities (Raven test (total score) and local connectivity strengths of the nodes in the brain network. 68 ROIs are reconstructed according to Desikan-Killiany Atlas [50]. The correlations are sorted from the highest to lowest. The P-FIT areas are highlighted with blue.

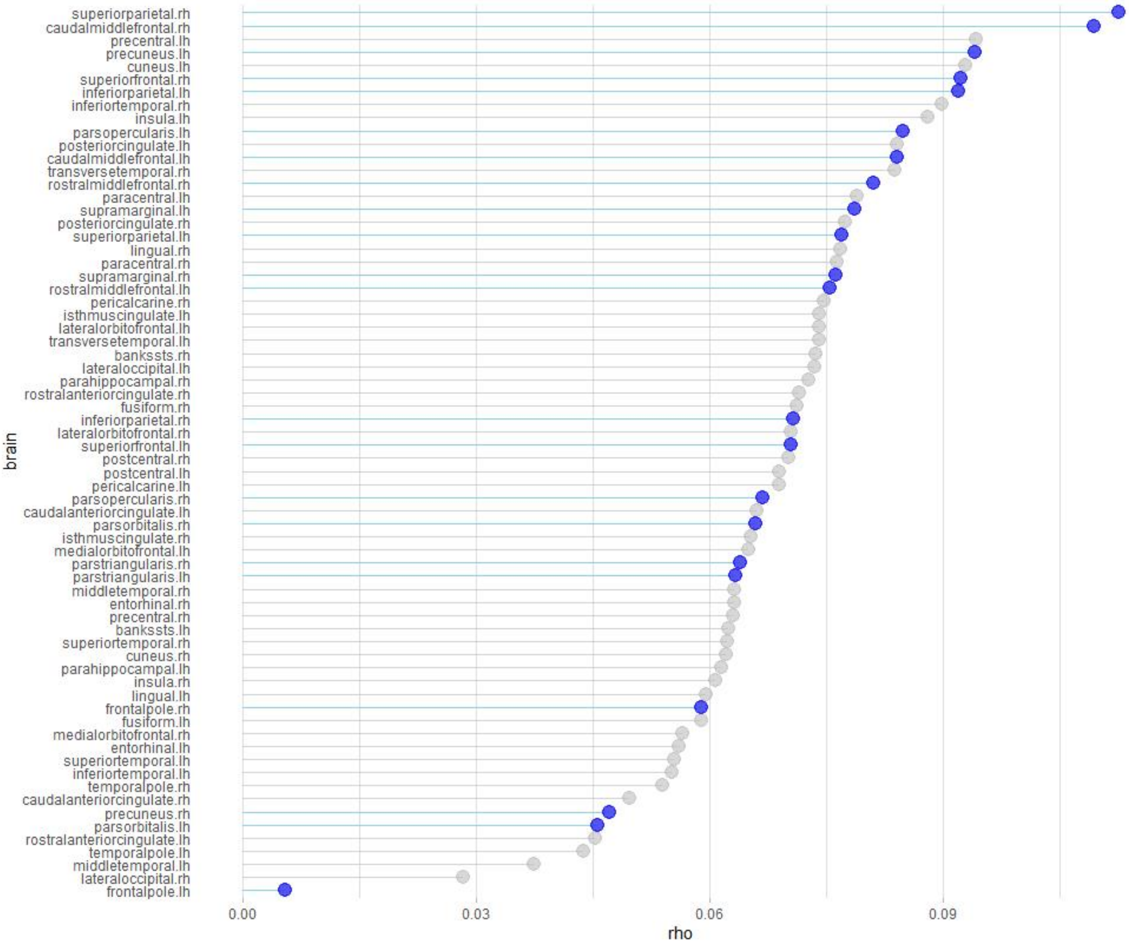

Figure S10. The relationship between verbal abilities (the test UIT1 ("Awareness") and local connectivity strengths of the nodes in the brain network. 68 ROIs are reconstructed according to Desikan-Killiany Atlas [50]. The correlations are sorted from the highest to lowest. The P-FIT areas are highlighted with blue.

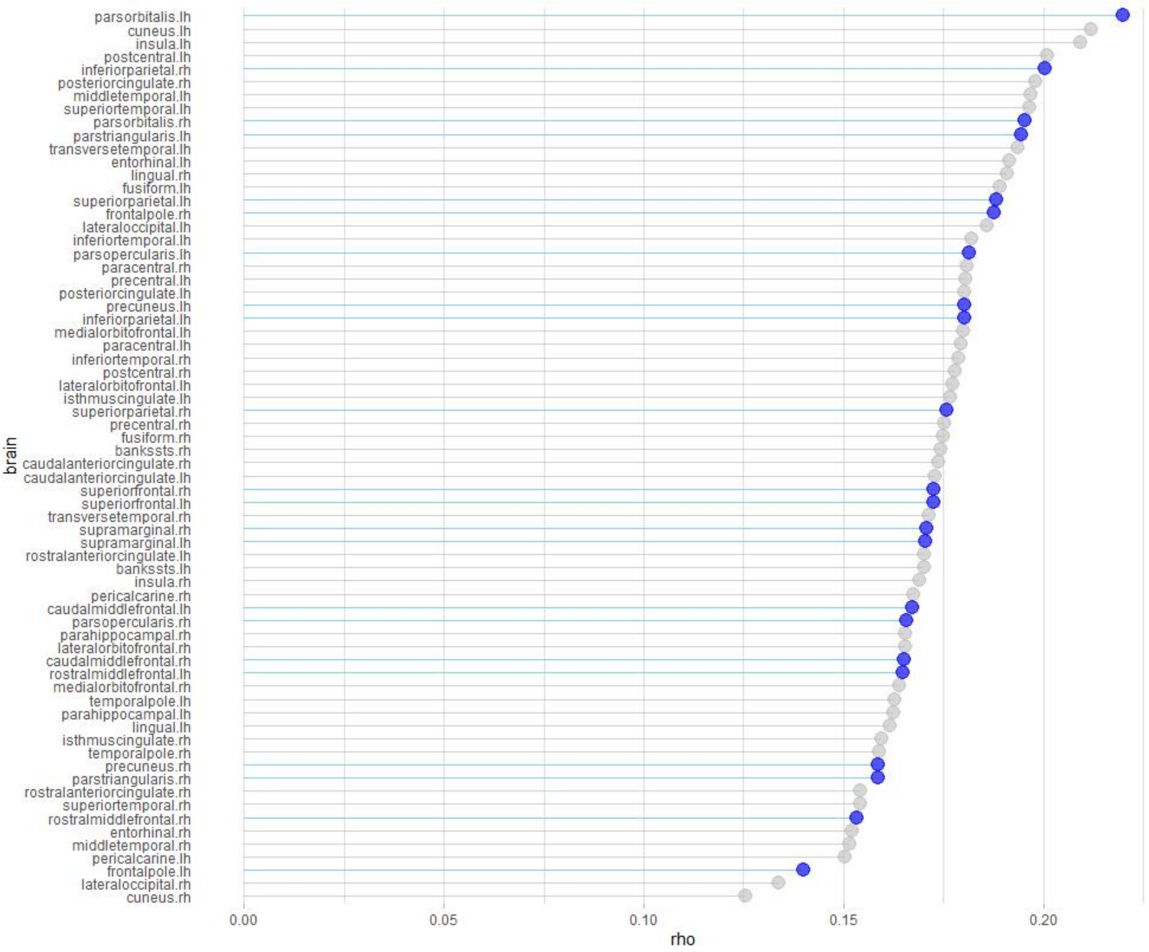

Figure S11. The relationship between verbal abilities (the test UIT5 ("Conclusions") and local connectivity strengths of the nodes in the brain network. 68 ROIs are reconstructed according to Desikan-Killiany Atlas [50]. The correlations are sorted from the highest to lowest. The P-FIT areas are highlighted with blue.

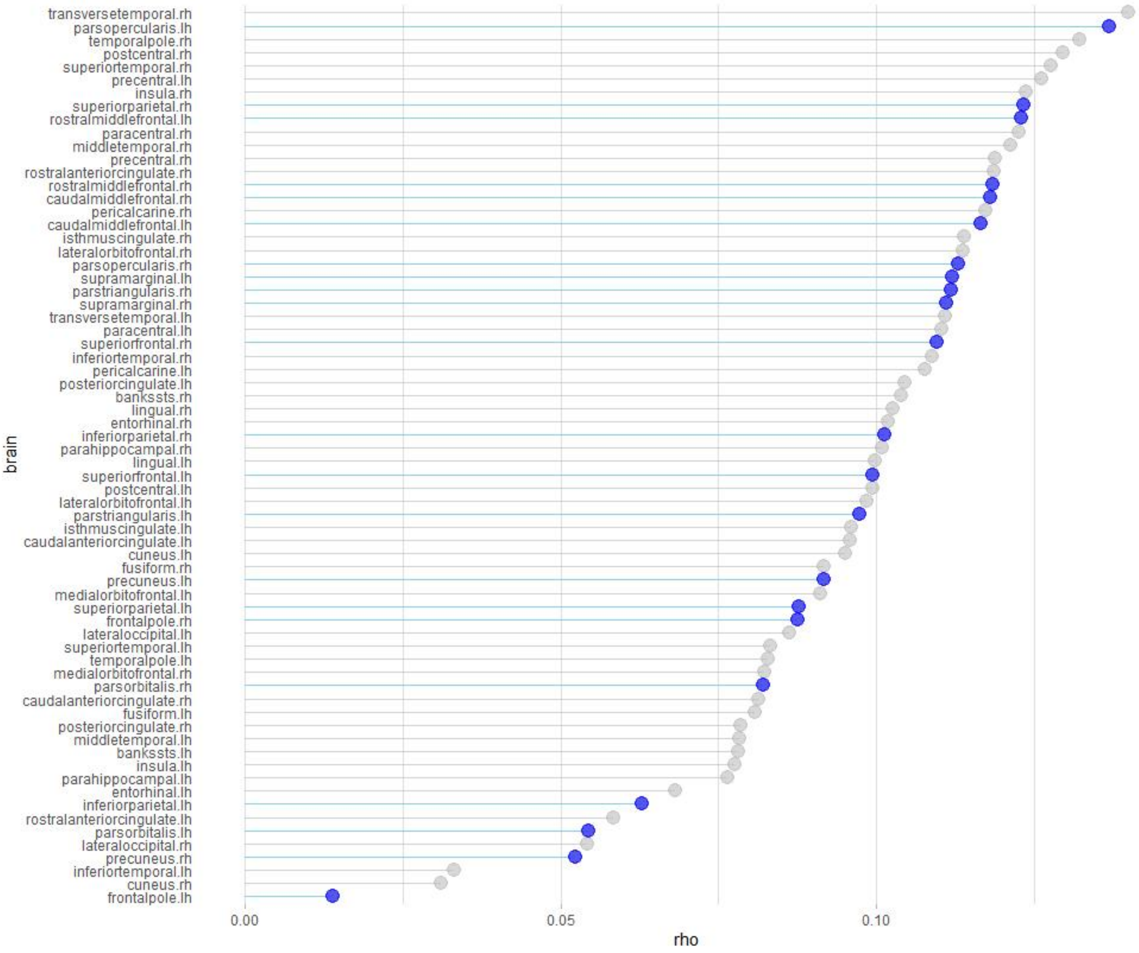

Figure S12. The relationship between verbal abilities (the test MyVocab) and local connectivity strengths of the nodes in the brain network. 68 ROIs are reconstructed according to Desikan-Killiany Atlas [50]. The correlations are sorted from the highest to lowest. The P-FIT areas are highlighted with blue
